# Prevention of Pain after Actual Cautery

**Published:** 1876-04

**Authors:** 


The Prevention of Pain After Application of the Actual Cautery—*By R. J. Levis, M. D.*—The increasing use of the actual cautery as a powerful surgical resource will render important a means of avoiding the suffering which, for a varying time, follows its local application. The practice of using the actual cautery seems to be now reviving, under the influence of some high authorities, and, perhaps, with some newly-developed views of its therapeutic action, especially with regard to its influence by peculiar impression on peripheral nerves.

The local anæsthetic action of carbolic acid has been for some time recognized by surgeons; but no important practical applications appear to have been deduced, and its great powers in that direction seem not to have generally impressed the profession.

I have been in the habit of taking advantage of this remarkable property of carbolic acid in my frequent resort to the actual cautery, with the result of the most complete avoidance of consequent suffering. My practice is to apply *pure* carbolic acid on and for a short distance around each point of application of the cautery, before the patient recovers from the influence of the general anæsthetic which has been used. For convenience of application, the crystals of carbolic acid are deliquesed by warmth, and the liquid applied with a brush. The part is then covered with any light dressing. Should pain recur after excessive or deep use of the cautery, the application may be renewed ; but I have not in my experience found such resort necessary.

Since the important fact of the local anæsthetic action of carbolic acid has been familiar to me, I have more frequently used the actual cautery in surgical practice than formerly, particularly in neuralgic suffering and in chronic painful affections of joints, and always with fredom from suffering and with satisfactory results.

I trust that the suggestions of the beneficent use of this familiar agent may increase the general practice of the powerful therapeutic resource of the use of the actual cautery, which seemed, indeed, for a long time, to be almost another of the "lost arts" of surgery.—*Med. Times.*

